# Supplementary material for: Symptom burden according to dialysis day of the week in three times a week haemodialysis patients
Source: PLoS One. 2022 Sep 27;17(9):e0274599. doi: 10.1371/journal.pone.0274599 (PMC9514641; doi:10.1371/journal.pone.0274599)
Supplement: S1 Table — (DOCX) [file pone.0274599.s001.docx]

**S1 Table: Symptom severity stratified by HD day (Dialysis Day of the week: HD1, HD2, HD3)**

|  | Mon/Tue/Sun (HD1) | | Wed/Thu/Tue (HD2) | | Fri/Sat/Thu (HD3) | | Total | |
| --- | --- | --- | --- | --- | --- | --- | --- | --- |
| Pain | 338 | 67.6% | 342 | 63.5% | 168 | 55.1% | 848 | 63.1% |
| Breathlessness | 264 | 52.7% | 311 | 57.2% | 167 | 55.5% | 742 | 55.1% |
| Weakness | 415 | 82.8% | 444 | 82.5% | 236 | 77.6% | 1095 | 81.5% |
| Nausea | 196 | 39.4% | 194 | 35.8% | 91 | 29.9% | 481 | 35.8% |
| Vomiting | 110 | 22.0% | 112 | 20.7% | 58 | 19.3% | 280 | 20.8% |
| Poor Appetite | 254 | 50.3% | 263 | 48.4% | 127 | 41.8% | 644 | 47.6% |
| Constipation | 162 | 32.3% | 182 | 33.6% | 91 | 30.1% | 435 | 32.3% |
| Sore Mouth | 243 | 48.3% | 267 | 49.1% | 147 | 48.4% | 657 | 48.6% |
| Drowsiness | 329 | 65.1% | 358 | 66.4% | 178 | 59.1% | 865 | 64.3% |
| Poor Mobility | 358 | 71.2% | 376 | 69.2% | 202 | 66.4% | 936 | 69.3% |
| Itchiness | 314 | 62.5% | 350 | 64.6% | 183 | 60.4% | 847 | 62.9% |
| Difficulty sleeping | 304 | 60.3% | 360 | 66.3% | 186 | 61.2% | 850 | 62.9% |
| Restless leg | 253 | 50.2% | 312 | 57.7% | 150 | 49.7% | 715 | 53.1% |
| Change in skin | 232 | 46.3% | 265 | 49.2% | 149 | 49.0% | 646 | 48.1% |
| Diarrhoea | 130 | 25.8% | 146 | 26.9% | 87 | 28.7% | 363 | 26.9% |
| Anxious | 243 | 48.1% | 254 | 46.7% | 123 | 40.5% | 620 | 45.8% |
| Depression | 223 | 44.2% | 267 | 49.2% | 140 | 46.1% | 630 | 46.6% |

**The observations from 4 time points (baseline, 6,12 and 18 months) were used to inform this table.**
