# Supplementary material for: Symptom burden according to dialysis day of the week in three times a week haemodialysis patients
Source: PLoS One. 2022 Sep 27;17(9):e0274599. doi: 10.1371/journal.pone.0274599 (PMC9514641; doi:10.1371/journal.pone.0274599)
Supplement: S2 Table — (DOCX) [file pone.0274599.s002.docx]

**S2 Table: Symptom score determined by baseline characteristics adjusted for Dialysis Day of the week (HD1, HD2, HD3)**

| **Symptoms** | **<40** | **>65** | **Female** | **Vintage <1 yrs** | **Vintage >5 yrs** | **Charlson 0** | **Charlson >5** |
| --- | --- | --- | --- | --- | --- | --- | --- |
| **Pain** | -0.18  (-0.5 to 0.15) | -0.35 **  (-0.5- to -0.15) | 0.19  (-0.01 to 0.38) | 0.06  ( -0.15 to 0.27) | 0.2  (0.01 to 0.39) * | -0.46 **  (-0.69 to -0.23) | -0.01  (-0.28 to 0.26) |
| **Breathlessness** | -0.09  (-0.39 to 0.21) | -0.1(-0.28 to 0.07) | -0.13  (-0.31 to 0.05) | 0.11  ( -0.07 to 0.29) | 0.19  (0.03-0.36)* | -0.26 *  (-0.48 to  -0.05) | 0.04  (-0.21 to 0.29) |
| **Weakness** | -0.03  (-0.35 to 0.29) | -0.39 **  (-0.58 to-0.21) | 0.1  (-0.09 to 0.28) | 0.02  (-0.17 to 0.22) | 0.07  (-0.11 to 0.25) | -0.14  (-0.36 to 0.09) | 0.23  ( -0.04 to 0.49) |
| **Nausea** | 0.06  (-0.21 to 0.32) | -0.27 **  (-0.43to-0.12) | 0.1  (-0.05 to 0.25) | 0.04  (-0.13to 0.21) | -0.04  (-0.19 to 0.11) | -0.09  (-0.28 to 0.09) | 0.11  (-0.11 to 0.32) |
| **Vomiting** | -0.005  (-0.22 to 0.210 | -0.2  (-0.34 to -0.1)** | 0.01  (-0.11 to 0.14) | -0.05  (-0.19 to 0.1) | 0.036  (-0.09to 0.16) | -0.11  (-0.25 to 0.04) | 0.05  (-0.12 to 0.23) |
| **Poor Appetite** | -0.16  (-0.45 to 0.13) | -0.3 **  (-0.47 to -0.13) | 0.15  (-0.03 to 0.32) | -0.07  (-0.25 to 0.11) | -0.07  (-0.23 to 0.1) | -0.06  (-0.26 to 0.15) | 0.33  (0.1 to 0.57)** |
| **constipation** | -0.25  (-0.53 to 0.02) | -0.19  (-0.34 to-0.03)* | 0.09  (-0.07 to 0.25) | 0.03  (-0.13 to 0.19) | -0.06  (-0.21 to 0.09) | -0.13  (-0.32 to 0.06) | 0.1  (-0.11 to 0.33) |
| **Sore mouth** | -0.13  (-0.41 to 0.16) | -0.06  (-0.23 to 0.1) | 0.14  (0.03 to 0.31) | -0.14  (-0.32 to 0.04) | -0.16  (-0.32 to 0.001) | -0.26 **  (-0.46 to -0.07) | 0.2  (-0.03 to 0.44) |
| **drowsy** | -0.31  (-0.61 to -0.005)* | -0.2  (-0.38 to -0.03)* | 0.005  (-0.17 to 0.18) | -0.04  (-0.24 to 0.15) | 0.05  (-0.12 to 0.22) | -0.2 *  (-0.44 to -0.02) | 0.24  (-0.01 to 0.49) |
| **Poor mobility** | -0.2  (-0.57 to 0.16) | -0.13  (-0.34 to 0.08) | 0.06  (-0.16 to 0.28) | 0.06  (-0.15 to 0.27) | 0.1  (-0.1 to 0.3) | -0.48 **  (-0.74 to-0.22) | 0.44 **  (0.14 to 0.74) |
| **itchy** | -0.005  (-0.34 to 0.330) | -0.1  (-0.29 to 0.1) | 0.006  (-0.19 to 0.21) | 0.27  (0.06 to 0.49)* | -0.05  (-0.24 to 0.14) | -0.016  (-0.25 to0.22) | 0.38  (0.1 to 0.65)** |
| **Diff sleep** | -0.19  (-0.55 to 0.17) | -0.51 **  (0-0.72 to-0.3) | -0.01  (-0.23 to 0.2) | 0.39  (0.18 to 0.6)** | 0.23  (0.03 to 0.43)* | -0.19  (-0.45 to 0.06) | 0.14  (-0.16 to 0.43) |
| **Restless leg** | 0.008  (-0.34 to 0.36) | -0.42 **  (-0.62 to-0.22) | 0.14  (-0.07 to 0.35) | 0.1  (-0.1 to 0.3) | 0.005  (-0.19 to 0.2) | -0.23  (-0.47 to 0.02) | 0.34  (0.05 to 0.63)* |
| **Change in skin** | -0.1  (-0.42 to 0.18) | -0.07  (-0.25 to 0.1) | 0.2  (0.05 to 0.4)* | 0.008  (-0.19 to 02) | -0.07  (-0.24 to 0.1) | 0.098  (-0.11 to 0.3) | 0.16  (-0.08 to 0.4) |
| **Diarrhea** | -0.2  (-0.46 to 0.04) | -0.07  (-0.22 to -0.08) | 0.05  (-0.1to 0.2) | 0.09  (-0.07 to 0.25) | 0.0001  (-0.14 to 0.24) | -0.08  (-0.26 to 0.09) | 0.1  (-0.11 to 0.3) |
| **Anxious** | 0.06  (-0.26 to 0.37) | -0.24 **  (-0.42 to-0.06) | 0.1  (-0.08 to 0.29) | -0.07  (-0.26 to 0.12) | -0.09  (-0.26 to 0.09) | -0.04  (-0.26 to 0.18) | 0.24  (-0.02 to 0.5) |
| **Depression** | -0.12  (-0.44 to 0.19) | -0.35 **  (-0.53 to-0.17) | 0.02  (-0.16 to 0.21) | 0.07  (-0.12 to 0.26) | 0.07  (-0.11 to 0.24) | -0.13  (-0.35 to 0.09) | 0.1  (-0.16 to 0.36) |

**The symptom outcomes were scored on a 0 (no symptoms) to 4 (overwhelming symptoms) scale. Results were described in effect size and (95% confident interval). Male was compared against female. Vintage <1yr and >5years was compared against vintage 1-5 years. Charlson index score 0 and >5 was compared against score 1-5. Age group <40 and >65 years was compared against age group 40-65 years. ** P <0.01, * P<0.05**
