# Supplementary material for: Symptom burden according to dialysis day of the week in three times a week haemodialysis patients
Source: PLoS One. 2022 Sep 27;17(9):e0274599. doi: 10.1371/journal.pone.0274599 (PMC9514641; doi:10.1371/journal.pone.0274599)
Supplement: S3 Table — (DOCX) [file pone.0274599.s003.docx]

**S3 Table: Baseline patient characteristics of participants who completed 1 instrument and >1 instruments throughout the study**

|  |  | Participants who completed 1 instrument Number(Percentage) | Participants who completed >1 instruments  Number(Percentage) |
| --- | --- | --- | --- |
| Age | Mean age | 64.81 | 62.86 |
| Sex | Male | 19(30.6%) | 185 (37.7%) |
|  | Female | 34 (54.8%) | 291(59.4%) |
|  | Missing | 9 (14.5%) | 14 (2.9%) |
| Charlson Comorbidity index score* | 0 | 15(27.8%) | 107 (23.9%) |
|  | 1-5 | 34(63%) | 273 (61.1%) |
|  | >5 | 5(9.3%) | 67 (15%) |
| Vintage (years on HD) | <1year | 13(28.9%) | 92(22.7%) |
|  | 1-5 years | 18 (40.0%) | 201(49.6%) |
|  | >5 years | 14 (31.1%) | 112(27.7%) |
| Ethnicity | White | 43 (69.4%) | 384 (78.3%) |
|  | Non-White | 10 (16.1%) | 83 (17%) |
|  | Missing | 9 (14.5%) | 23 (4.7%) |

* **Higher Modified Charlson score indicates high comorbidities.**
