# Supplementary material for: Symptom burden according to dialysis day of the week in three times a week haemodialysis patients
Source: PLoS One. 2022 Sep 27;17(9):e0274599. doi: 10.1371/journal.pone.0274599 (PMC9514641; doi:10.1371/journal.pone.0274599)
Supplement: S4 Table — (DOCX) [file pone.0274599.s004.docx]

| 5 common symptoms | severity | Symptom severity at baseline | |
| --- | --- | --- | --- |
|  |  | Completed 1 instrument | Completed >1 instruments |
| Weakness or lack of energy | None | 12.3% | 17.2% |
|  | Mild | 26.3% | 24.3% |
|  | Moderate | 40.4% | 31.4% |
|  | Severe | 19.3% | 21.1% |
|  | Overwhelming | 1.8% | 6.1% |
| Poor mobility | None | 27.6% | 30.5% |
|  | Mild | 29.3% | 19.6% |
|  | Moderate | 27.6% | 24.8% |
|  | Severe | 13.8% | 19.6% |
|  | Overwhelming | 1.7% | 5.4% |
| Drowsiness | None | 37.9% | 31.7% |
|  | Mild | 24.1% | 29.4% |
|  | Moderate | 32.8% | 27.7% |
|  | Severe | 5.2% | 8.8% |
|  | Overwhelming | 0.0% | 2.3% |
| Difficult sleeping | None | 31.0% | 33.6% |
|  | Mild | 25.9% | 21.3% |
|  | Moderate | 20.7% | 24.4% |
|  | Severe | 15.5% | 14.2% |
|  | Overwhelming | 6.9% | 6.5% |
| Pain | None | 38.6% | 37.5% |
|  | Mild | 29.8% | 22.1% |
|  | Moderate | 19.3% | 24.4% |
|  | Severe | 12.3% | 13.1% |
|  | Overwhelming | 0.0% | 2.9% |

**S4 Table: 5 common symptoms comparing their severity at baseline for Participants who completed only 1 instrument and >1 instruments throughout the study**
