# Supplementary material for: Symptom burden according to dialysis day of the week in three times a week haemodialysis patients
Source: PLoS One. 2022 Sep 27;17(9):e0274599. doi: 10.1371/journal.pone.0274599 (PMC9514641; doi:10.1371/journal.pone.0274599)
Supplement: S5 Table — (DOCX) [file pone.0274599.s005.docx]

**S5 Table**: **Multivariable mixed effects linear regression comparing each hemodialysis day after long break, each HD day from HD1, HD day vs non-HD day**

| **Symptoms** | Increasing day from HD1 | HD1 vs HD2 | HD1 vs HD3 | HD vs Non-HD |
| --- | --- | --- | --- | --- |
| **EQ5D5L** | 0.01 (-0.01 to 0.02) | -0.01 (-0.03 to 0.02) | 0.02 (-0.01 to 0.05) | -0.06 (-0.09 to -0.03)* |
| **Pain** | -0.01 (-0.09 to 0.07) | 0.07 (-0.07 to 0.21) | -0.04 (-0.2 to 0.13) | 0.11 (-0.05 to 0.27) |
| **Breathlessness** | 0.04 (-0.03 to 0.11) | 0.17 (0.05 to 0.27)* | 0.04 (-0.1 to 0.18) | 0.09 (-0.05 to 0.23) |
| **Weakness** | 0.02 (-0.06 to 0.1) | 0.2 (0.07 to 0.33)* | -0.01 (-0.16 to 0.14) | 0.13 (-0.02 to 0.28) |
| **Nausea** | -0.03 (-0.09 to 0.04) | 0.04 (-0.07 to 0.16) | -0.07 (-0.2 to 0.07) | 0.11 (-0.03 to 0.24) |
| **Vomiting** | 0.01 (-0.04 to 0.07) | 0.04 (-0.05 to 0.14) | 0.02 (-0.09 to 0.13) | 0.06 (-0.05 to 0.16) |
| **Poor Appetite** | -0.03 (-0.1 to 0.04) | 0.08 (-0.04 to 0.19) | -0.09 (-0.23 to 0.05) | 0.2 (0.06 to 0.34)* |
| **constipation** | 0.01 (-0.06 to 0.07) | 0.05 (-0.06 to 0.16) | 0 (-0.13 to 0.13) | 0.19 (0.06 to 0.31)* |
| **Sore mouth** | 0.06 (-0.01 to 0.13) | 0.07 (-0.05 to 0.18) | 0.11 (-0.03 to 0.25) | 0.01 (-0.12 to 0.15) |
| **drowsy** | -0.03 (-0.1 to 0.05) | 0.01 (-0.12 to 0.13) | -0.06 (-0.22 to 0.09) | 0.02 (-0.12 to 0.17) |
| **Poor mobility** | -0.01 (-0.09 to 0.07) | 0.02 (-0.11 to 0.16) | -0.02 (-0.18 to 0.15) | 0.18 (0.03 to 0.33)* |
| **itchy** | -0.04 (-0.13 to 0.04) | -0.08 (-0.22 to 0.06) | -0.08 (-0.25 to 0.09) | 0.08 (-0.08 to 0.24) |
| **Difficult sleep** | 0.06 (-0.02 to 0.14) | 0.23 (0.09 to 0.36)* | 0.08 (-0.08 to 0.25) | 0.2 (0.04 to 0.36)* |
| **Restless leg** | 0.1 (0.02 to 0.18)* | 0.18 (0.05 to 0.32)* | 0.17 (0.01 to 0.33)* | 0 (-0.15 to 0.15) |
| **Change in skin** | 0.08 (0.01 to 0.16)* | 0.13 (0.01 to 0.26)* | 0.15 (0 to 0.3)* | 0.14 (-0.01 to 0.29) |
| **Diarrhea** | 0.04 (-0.02 to 0.11) | 0.08 (-0.02 to 0.19) | 0.08 (-0.05 to 0.2) | -0.05 (-0.17 to 0.07) |
| **Anxious** | 0.02 (-0.06 to 0.09) | 0.01 (-0.12 to 0.13) | 0.03 (-0.12 to 0.18) | 0.11 (-0.03 to 0.25) |
| **Depression** | 0.03 (-0.04 to 0.1) | 0.12 (0 to 0.24) | 0.03 (-0.11 to 0.18) | 0.17 (0.03 to 0.31)* |

**Models adjusted for age, sex, comorbidity (Charlson 0, 1-5, >5), time on dialysis (<1 year, 1-5 and >5 years)**

**Results were described in effect size (a value of 1.0 would represent a change from none to mild or severe to overwhelming) and 95% confident interval.**

**The symptom outcomes were scored on a 0 (no symptoms) to 4 (overwhelming symptoms) scale.**

*** P<0.05**
