# Supplementary material for: Symptom burden according to dialysis day of the week in three times a week haemodialysis patients
Source: PLoS One. 2022 Sep 27;17(9):e0274599. doi: 10.1371/journal.pone.0274599 (PMC9514641; doi:10.1371/journal.pone.0274599)
Supplement: S6 Table — (DOCX) [file pone.0274599.s006.docx]

S6 TABLE: STROBE check list for observational study

| Topics | Item No. | Checklist Item | Page  No. |  |  |  |
| --- | --- | --- | --- | --- | --- | --- |
| **Title and abstract** | 1 | (*a*) Title clearly identified the aim of the study | 1 |  |  |  |
|  |  | (*b*) Structured summary of trial design, methods, results, and conclusions | 2 |  |  |  |
| Introduction | | | |  |  |  |
| **Background/****rationale** | 2 | The scientific background and rationale for the investigation being reported | 3 |  |  |  |
| **Objectives** | 3 | Specific objectives, including any prespecified hypotheses | 3-4 |  |  |  |
| Methods | | | |  |  |  |
| **Study design** | 4 | Key elements of study design, relevant dates, including periods of recruitment, exposure, follow-up, and data collection | 4-5 |  |  |  |
| **Setting** | 5 | Study setting and involved dialysis centres | 4-5 |  |  |  |
| **Participants and data collection** | 6 | Eligibility criteria for participants  locations where the data were collected | 4-5 |  |  |  |
| **Variables** | 7 | Covariate adjustment variables data collection | 5 |  |  |  |
| **Study size** | 8 | How sample size was determined. Method of calculation and relevant parameters with sufficient detail so the calculation can be replicated. Assumptions made about correlations between outcomes of participants from the same cluster. | 6 |  |  |  |

| **Statistical** **methods** | 9 | (*a)*Statistical methods used to compare symptom prevalence stratified by dialysis day of the week and mixed effect linear regression to detect change in symptom severity in relation to dialysis day of the week | 6-7 |  |  |  |  |
| --- | --- | --- | --- | --- | --- | --- | --- |
|  |  | (*b* Methods for additional analyses, such as subgroup analyses, sensitivity analyses, and adjusted analyses | 6-7 |  |  |  |  |
|  | | | | |  |  | Results |
| **Participants** | 10 | (a) a flow diagram | Fig 1 |  |  |  |  |
| **Descriptive** **data** | 11 | (a) Characteristics of study participants | Page 7, Table 1 |  |  |  |  |
| **Outcome data** | 12 | Symptom prevalence and its association with patient’s characteristics | 8-10, Table 2, S1 and S2 Table |  |  |  |  |
| **Main results** | 13 | Effect of symptom burden and change in symptom score according to Dialysis Day of the Week | 10-11, Fig 3, S5 Table |  |  |  |  |

| Discussion | | | | |
| --- | --- | --- | --- | --- |
| **Key results and interpretation** | 14 | Summary of key results with reference to study objectives and overall interpretation of results considering objectives | 12 |  |
| **Limitations** | 15 | Limitations of the study and magnitude of any potential bias | 12-13 |  |
| **Generalisability** | 16 | Generalisability (external validity) of the study results | 13 |  |
| Other information | |  | | |
| **Funding** | 17 | Source of funding and the role of the funders for the present study and for the original study on which the present article is based | 4 and Cover letter |  |
