# Supplementary material for: Symptom burden according to dialysis day of the week in three times a week haemodialysis patients
Source: PLoS One. 2022 Sep 27;17(9):e0274599. doi: 10.1371/journal.pone.0274599 (PMC9514641; doi:10.1371/journal.pone.0274599)
Supplement: Attachment — Submitted filename: Response to reviewer(s).docx [file pone.0274599.s008.docx]

**REBUTTAL LETTER FOR SUBMISSION OF REVISED MANUSCRIPT**

To The Editor in Chief,

PLOS ONE Journal	Date: 11/08/2022

We are grateful for your and the reviewers’ comment on the manuscript “**Symptom Burden According To Dialysis Day Of The Week In Three Times A Week Haemodialysis Patients** “. We’ve revised and modified the manuscript according to reviewers’ critiques. In the following, we address each of their comment.

Journal Requirements:

Please review your reference list to ensure that it is complete and correct. If you have cited papers that have been retracted, please include the rationale for doing so in the manuscript text, or remove these references and replace them with relevant current references. Any changes to the reference list should be mentioned in the rebuttal letter that accompanies your revised manuscript. If you need to cite a retracted article, indicate the article’s retracted status in the References list and also include a citation and full reference for the retraction notice.

I’ve reviewed the reference list and they are complete. There was no citation of retracted papers.

Response to reviewer 2

1. Your response to my point 11 ("No mention of HRQoL in the study’s aim") is unclear: what does 'effect of symptom burden as a mean of HRQoL' mean? I strongly recommend you say something about the role/importance of HRQoL at the end of the introduction, so that this doesn't come as a surprise to readers when they get to the Methods and Results (e.g. that it was a secondary outcome of interest?)

We’ve added the role of HRQoL in this study. (line number 83-88)

***“****HRQoL has been shown to be a predictor of morbidity and mortality in haemodialysis patients (14)(15) and HRQoL measures play an important role in evaluating cost effectiveness of treatment. Failure to account for any underlying differences in symptom severity due to the day of instrument completion in relation to the dialysis schedule could bias the impact and effectiveness of interventions for symptoms and HRQoL, which have been prioritised by the patients and clinicians(2) and could lead to failure of new treatment or interventions to be approved.”*

2. Your response to my point 14 (“provide information on how characteristics of people compared between those in- and excluded in the analysis”) is adequate. However, I suggest you include the relevant analyses/tables as supplementary materials, so that interested readers also have access to this information.

We’ve added this in result section and provided supplementary tables. (line number 234-237 and Supplementary table 3 and 4).

*“There were 62 participants who completed only one instrument throughout the study period. Comparing this cohort with patients who completed the instruments more than once in follow up period, patient characteristics and severity of symptom burden at baseline were similar (S3 Table, S4 Table).”*

3. Your response to my point 16 (“State the level at which you considered a finding statistically significant”) is unclear: it’s hard to follow your argument for why statistical significance is (or isn’t?) relevant. But regardless of what you decide to use as a threshold, please clarify for readers in the manuscript (and not just for me in the response letter) what your definition of ‘statistical significance’ is and how you accounted for multiple testing (or why this is not required).

We’ve clarified this in method as well as in discussion section. (line number 178, 309-313)

*“P value < 0.05 was considered the threshold for statistical significance.”*

*“**We performed multiple testing with different variables with statistical significance considered as a p value less than 0.05, and concluded adjustment for dialysis day of the week is not required as most of the results were non-significant. Correction of p values for multiple testing would further reduce significance, not altering our conclusions, and is therefore is not required.”*

4. There are several grammatical/style errors throughout the newly added sections in the abstract and main text - the manuscript therefore requires one, last thorough round of text editing by a native English speaker prior to submission.

We hope we have identified and corrected the errors.
